# Systems Approaches to the Eukaryotic Stress Response

**DOI:** 10.1371/journal.pcbi.1004757

**Published:** 2016-03-10

**Authors:** Christine Vogel

**Affiliations:** Center for Genomics and Systems Biology, Department of Biology, New York University, New York, United States of America; EMBL Heidelberg, GERMANY

## How the Lab Started

**Start of the lab:** 2011

**Size of the lab:** Two postdocs, three PhD students, master and undergraduate students, and one (post)dog

**Research field:** Systems biology; proteomics; integrative, dynamic gene expression regulation

I joined New York University (NYU) in 2011 because it offered a combination of scientific excellence, institutional support, awesome colleagues, and living conditions that were the right fit for me. I have now been an assistant professor in the Department of Biology’s Center for Genomics and Systems Biology for five years, and I am proud to have a great lab up and running.

## Scientific Mission and Funding

Our lab uses a combination of proteomics methods, computational and statistical analysis, and molecular tools to understand protein expression in response to environmental stress. We are interested in very fundamental questions about the relative contributions of transcription and translation to setting final protein concentrations [1–3], but we also use the tools that we develop to help our understanding of human diseases. Because we employ proteomics as a tool, we are highly collaborative and work with several different groups. For example, in collaboration with one of my colleagues, we investigate mRNA and protein expression changes in different types of motor neurons responding to stress of the endoplasmatic reticulum. Moreover, in a different line of research, we are characterizing a new function for protein ubiquitination under oxidative stress that we recently discovered [4].

With respect to funding, I am still learning how to best “sell” my work. My advice for successful grant applications is to emphasize the field in which you are established as an expert and to look for a funding opportunity that corresponds to that area. Because of the fundamental character of many of our projects and my track record in method development, my most successful grant application was indeed not when I proposed disease-related work but instead was when I requested funding for the development of a new statistical tool to analyze time-series proteomics and transcriptomics data.

## Short Stories That Will Leave You Smiling, Crying, and Thinking

I had very good mentors during my scientific career, and one of my ongoing goals and passions is to pass that on and be a good mentor myself. Earlier last year, my mother passed away after a very aggressive illness. Helping to take care of her, in addition to my own serious health challenges, had made running a lab highly stressful and demanding during that time. Yet, despite reaching my limits, I always tried to leave my own issues at home when arriving at work; I consider it to be my job as a leader to take care of my lab and my lab members. But when I came back after my mother’s funeral, I found a gigantic bouquet of flowers and a card in my office. My postdocs and students had signed the card with notes that brought tears to my eyes, many of which conveyed how they appreciated that I was always there for them, how they liked to be in an encouraging environment, and how they admired that I could keep a sense of humor even in tough times (which makes me particularly proud since I am German!). It showed to me that I had achieved that for which I had worked so hard: to be a good captain of our ship.

“I found a gigantic bouquet of flowers and a card in my office”

## Time Management and Delegation of Work

Sometimes, I see some of my colleagues struggling to transition from being a postdoc in complete control of their projects to the uncertainty that comes with being a principal investigator (PI), particularly in regards to the progress of research, and the daily frustration that occurs when things do not move as quickly as they would like. It is easy to think that the job will be done more efficiently if we do it ourselves, but I am happy to say that I have not had problems with delegating work. It probably helps that I, myself, do not like to do benchwork. In my opinion, an important aspect of being able to delegate is to let go of perfectionism. That is my advice: let go of perfectionism. Yes, you are on a tenure clock and under a lot of pressure—but the world will not end if something is not done right on time, in your way. Even if others might change some details and perform the task differently than the way you originally imagined, it will be fine in the end.

I think another important skill to strive for is the ability to prioritize well. My senior colleague once told me that more important than learning to delegate your work is to learn what to delegate and what to do yourself. Even after delegating some tasks, you will find that your to-do list is much longer than what you will ever be able to manage. So, instead, I suggest spending time on evaluating what is really important and what is urgent (with a deadline). From there, decide what to do next and which tasks can wait or can be done by others. Work on that one thing and try and ignore the other tasks. One item at a time.

Finally, when you start a lab, you are often warned that you will rarely find time for creative research, as you will be busy writing grants, teaching, mentoring students, going to meetings, and doing administrative work. Try out a few methods to free up time for research. For example, my most productive time is in the morning; hence, I try to schedule all meetings during the afternoon. If I can, I also work from home in the morning so that I am not interrupted by anything or anyone else. Scheduling helps me a lot, too: I meet with every lab member once a week, so interruptions are less likely. I am not a fan of unscheduled phone calls for the same reason; I find them very disruptive. And currently, I am trying a new method to increase the time I spend on reading literature, thinking, and writing papers. Each weekday morning, if possible, I spend one hour reading and two to three hours writing. It is an illusion to try and get “the other things” out of the way before reading or writing my own papers, so I am trying to schedule time for it, even if “the other things” have not been done yet. Try a few of these tricks and see what works for you.

“Each weekday morning, if possible, I spend one hour reading and two to three hours writing”

## A Day in the Lab

I am a morning person and usually get up well before 7 am. If I can, I like to work from home in the morning and arrange for all my meetings to be during or after lunch. Having gotten something done in the morning lets me be relaxed enough to not rush through a creative discussion that pops up. I do not believe in making my lab members arrive and leave at a specific time—I think that that is unrealistic for biological experimentation and is counterproductive. During our weekly meetings, I can see the progress of a project—I do not care when that progress happened. However, my lab members are expected to be available during work hours via email or in person and to let me know in advance about their vacation time.

## Team Building and Retreats

Our department has an annual retreat that is a day of science and scientific fun, a great way to get to know colleagues and students in an informal atmosphere. The retreat is organized by the graduate students and is an essential component of our department’s culture.

With regards to my lab, each summer we have an annual picnic, and we have also gone skiing together. I find myself spending a surprisingly large amount of time thinking about team building, and I know I am not the only PI doing that. Do I take my students out for lunch or not? Should we celebrate our lab members’ birthdays or not? Should we all go for beer, even though most of us do not really like beer? I think all of this is up to personal taste, and there is more than one right answer. For example, we do celebrate our lab members’ birthdays, with a card and a cake, and everyone looks forward to that. Occasionally, we go for ice cream or hot chocolate (depending on the season). Again, I think the key to team building is to let go of your perfectionism and do what is right for you—even if you might not be the “perfect” team leader, you will be doing the best job you can, and it will be fine. If you do not like beer, no need to go for beer.

“I find myself spending a surprisingly large amount of time thinking about team building, and I know I am not the only PI doing that”

Within this topic, I would also like to share an interesting thought by a colleague of mine. When you hire someone for your lab, for example, a new postdoc, consider this: if the postdoc is just not as good with his or her research, then you lose a postdoc. That might hurt, because it costs you money. However, what is even worse is if the person is bad for the group—then you lose the entire team. I try to keep that in mind when I hire people.

“If the person is bad for the group—then you lose the entire team”

Finally, I strongly believe that showing your human side is part of good leadership and team building. For example, my lab knows how much I love my dog, and he is a fully accepted lab member, even listed on our website. Upon seeing another lab’s t-shirts, I was immediately envious and told my lab we needed one too. One of my postdocs got as excited as me about the idea, and together, we designed a new lab logo and finally printed Vogel lab t-shirts, which we proudly wear at many occasions.

## Project Management

Project management is a continuous act of juggling between people, power, time and effort, potential impact of the work, progress, and of course, funding. How do you balance creativity with strategy? Breadth in research topics and depth in analysis? Collaborative work and your lab’s own projects? Working on the one big high-impact paper that would not be finished in five years versus getting smaller projects published in more specialized journals? Again, I believe in letting go of perfectionism, as the list of attractive projects is likely longer than what you and your team can handle, and frankly, there is no way you can do everything. Prioritization and a healthy balance are the key. While you obviously want to produce quality work, a published medium-impact paper is better than a high-impact paper that is never finished. For example, after we published our ubiquitin work in a high-impact journal [4], I decided that it was time to work on a few smaller, “forgotten” projects. Without aiming for high-impact journals, this process was very productive, and the people involved were happy to boost their curricula vitae with a few more papers, even if they did not come from the top journals.

Further, I try to distribute projects according to the individual’s needs and capabilities. For example, my postdocs would usually work on one safe, bread-and-butter project and one bigger and riskier project of potentially high impact. My PhD students work on projects that are somewhere between being safe and high impact. In addition, lab members participate in each other’s projects and help out with collaborative work, which ensures faster progress but also provides a safety net in case some projects fail.

Handling collaborations outside the lab is one of the trickiest parts of project management for me. Proteomics is not an easy technique but highly suited to collaborations. While many collaborative ideas are fantastic on paper, we can often not predict the outcome. However, trying something new takes one person several days or weeks of work, and if the outcome is negative and there never is a paper, this process gets frustrating. Over the years, I have become much more careful with accepting collaborations. That is hard, as of course many projects are very interesting. But once you sit down with the potential collaborator and go through the effort, time, and costs of even pilot studies, it becomes clearer if the project is feasible, and it often turns out that working with us might not be the best solution to the problem.

“Handling collaborations outside the lab is one of the trickiest parts of project management for me”

Some people recommend agreeing on the author order in collaborative projects right at the beginning, which I sometimes find difficult to do, simply because it is often unclear in which direction the story will develop. We just had a case in which two postdocs on either side of a collaboration contributed high-quality work but could not agree who would be the first-first and who would be the second-first author on the joint paper. The best solution for this problem was to split the paper into two.

Further, part of management is to let go of unsuccessful projects, which is a very difficult task. Of course, I wish I had the wisdom to foresee failure long before it happens. Some projects do not die officially but end up “on ice” forever, as they never make it to the top of the priorities list. Other projects I officially close. In a recent case, we tried for two years to isolate exosomes for proteomics work but then found out that the system was inherently not suited for this type of analysis, and making progress on the work would have required enormous additional efforts. The postdoc who worked on this project and I had many conversations about it and finally set a deadline: if we did not see a publishable result by this date, we would give up. Of course, that was disappointing, but again, accepting imperfection, which in this case looks like failure, is what I embrace. What helped the postdoc was that long before we stopped the project, we had started to work on other projects, which then led to two publications. Balancing risky work (that can fail) with safe (but possibly less exciting) projects is what prevented disappointment and frustration for all parties. And for me personally, I found it enormously helpful to have a mission statement ready at hand—a couple of sentences describing the overall goal of my research. Having this helps me to let go of work that does not fit.

Finally, I suggest accepting that prioritizing your work is an ongoing process. Priorities are constantly changing, so much so that on a weekly basis, I step back and decide where to focus my attention next: “Which project is most urgent and most important right now?” Further, accept that every project is different and every problem asks for a different solution. As a leader, it is your job to balance all the factors that are involved while maintaining productive relationships within and outside the lab. Seek out resources around you that can help. For example, I asked other faculty for advice on a difficult authorship discussion. Or, for NYU Biology, we run “Faculty Lunches,” during which one of us discusses projects or a grant proposal that we are working on and the rest of us brainstorm the major points that should be prioritized. For big proposals and strategic planning, such meetings are very useful. If such a thing does not exist in your department, organize it yourself—invite a colleague for lunch and talk!

## Motivation

Of course, a big motivation for your lab members should be your own enthusiasm about your research. There are other motivation techniques, like setting internal deadlines or celebrating milestones, which may or may not be your style. Mass spectrometry requires quite a bit of training and technical knowledge, so whenever a new lab member is intimidated by the amount of information that needs to be acquired, I tell them that I do (almost) not care about how much or how little they know about mass spectrometry. It is something they can learn. But I do remind them that there is one thing I cannot teach them: being motivated to learn. Motivation is the key, and your task as a group leader is to stay on top of everyone’s performance. At each of our weekly one-on-one meetings, I monitor a person’s progress, trying to detect issues as early as possible. Listening is much more important than talking: if you detect a lack of motivation, rather than scolding, ask the student/postdoc what the reason might be. The answer might often surprise you.

“If you detect a lack of motivation, rather than scolding, ask the student/postdoc what the reason might be. The answer might often surprise you”

## International Experience

I love the international character of our scientific world. Throughout my career, I spent research time in Germany, England, Texas, and now in New York. Our recently accepted paper has ten co-authors on it, and none of us are native English speakers. My lab has, apart from myself, South American, Indian, Chinese, Spanish, and American members—so, we are quite diverse. That we have the chance to travel, to live in different countries, and to meet people from different cultures is one of the exciting aspects of academia and one of the bonuses we get for our work. In my view, we should embrace these differences and learn as much as we can from each other. And if worse comes to worst, my office library has a series of short books entitled “A Xenophobe’s Guide to…[insert culture here],” which are fun little guides to different cultures.

Of course, handling different cultures also brings extra challenges to leadership, requiring sensitivity, patience, diplomatic skills, and most importantly, communication. More than ever, it is important to find out the “why” behind incidents, rather than getting mad at an issue. For example, one of my lab members was always working very hard, doing everything I said but not a single thing beyond this. I got quite upset about this, as I felt I was micromanaging the project. Finally, I found out why this was happening: in the person’s culture, you follow your leader’s instructions precisely, and thinking (or working) on your own is very much discouraged as it’s a form of disrespect. Once I realized this, it was much easier to talk about this difference and to come up with constructive solutions. Listening (instead of talking) and asking questions (instead of placing blame) is very important, and you will be surprised to see what you learn.

## Advice to a Beginning Researcher and PI

If you are a student, pay attention to finding a good mentor. You might look at your potential PI’s publication record, his or her funding situation, and of course, whether the research topic is interesting to you, but finding a supportive mentor is equally important, especially at the beginning of your career. Everyone is different, so you want to find the mentor with whom you will work well. Do you want structured meetings or to just randomly drop by your PI’s office? Do you want to have help with every step of your project or rather be allowed to explore your own ideas? Do you know what skills you want to develop during your PhD and what you want to do afterwards? Does your PI know and help you with your plans? For example, during my PhD, I had two advisors, one senior researcher who had all the wisdom and insights from working for many years in the field and having seen many people come and go and one junior advisor who would invite me to play basketball but also push for papers to be finished. The balance between the two of them was perfect for me, and I appreciate how both challenged me to grow but also demonstrated their commitment to helping me become the researcher that I am.

One of the first lessons I learned as a young PI was that there are several different ways to run a lab, and there is more than one right way. Think of what you liked or disliked in the labs you have worked in and how you want to run your lab. Do you like to be hands-on or hands-off? Do you want to have weekly one-on-one meetings with everyone or not? Do you like to let your students try new things independently, or rather, do you want to manage every step? These are questions worth pondering before you start your lab, but keep in mind, these decisions may not be applicable to every one of your lab members. For example, with some of my students, I meet on a weekly basis and we end every meeting with a precise list of things to work on during the next week. Other lab members, such as my postdocs, meet with me less regularly, as they work more independently.

Three years into running my lab, I attended a short course at Cold Spring Harbor Laboratory on lab management modeled after a book called *Lab Dynamics*: *Management Skills for Scientists* (Carl M. Cohen and Suzanne L. Cohen). I wish I had taken that course earlier. I have been trained as a scientist, and I had thought a lot about mentoring, but suddenly, I had to have skills that did not come as easily: being a visionary, a manager, a team leader, a diplomat, a strategist, and sometimes a dictator. These are all skills that you can learn, but getting some training, especially through a course like this, helps to avoid having to reinvent the bicycle every single time.

And a final note on what I learned while being a PI: you can read all of the lab management books you want, read interviews like this, take a course, talk to your colleagues, or meditate over how you should run a lab, but in the end, it is always good to, at least every now and then, ignore all of that advice and just go for it. This is your chance to create something new and exciting that is entirely yours, and it is your chance to change the world a tiny little bit. So, enjoy!

“This is your chance to create something new and exciting that is entirely yours”

**Image 1:**
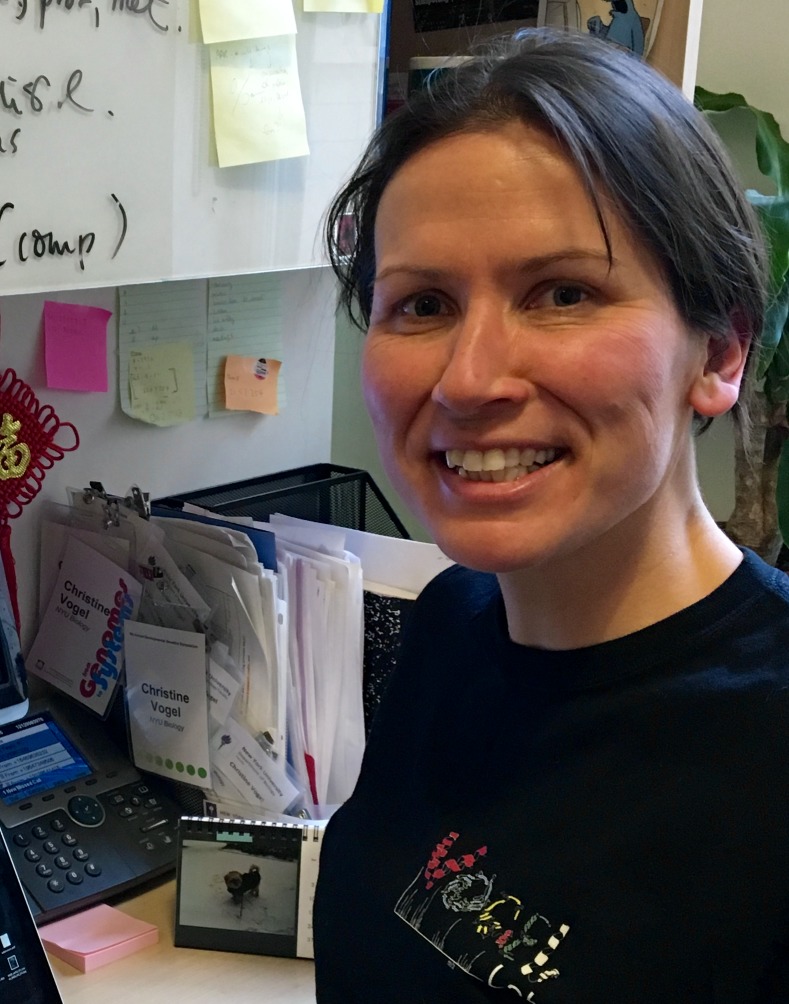
Christine Vogel. Photo courtesy of Christine Vogel.
